# B-Cell Activating Factor Predicts Acute Rejection Risk in Kidney Transplant Recipients: A 6-Month Follow-Up Study

**DOI:** 10.3389/fimmu.2019.01046

**Published:** 2019-05-15

**Authors:** Xu-Zhen Wang, Zhen Wan, Wu-Jun Xue, Jin Zheng, Yang Li, Chen Guang Ding

**Affiliations:** ^1^Critical Care Medicine, First Affiliated Hospital of Nanchang University, Nanchang, China; ^2^Department of General Surgery, First Affiliated Hospital of Nanchang University, Nanchang, China; ^3^Department of Kidney Transplantation, Hospital of Nephropathy, First Affiliated Hospital of Xi'an Jiaotong University, Xi'an, China

**Keywords:** BAFF, kidney transplantation, acute rejection, biomarker, C4d

## Abstract

B cell activating factor (BAFF) belonging to TNF family is a cytokine that enhances B-cell proliferation and differentiation. Recently, It has been suggested that BAFF might be a potential therapeutic target for treating autoimmune disease. However, the relationship between BAFF and allograft rejection is controversial, and the clinical significance of BAFF in predicting allograft rejection need to be further explored. We conducted 6-month follow-up study to confirm the hypothesis that BAFF might be a risk factor for predicting acute rejection in kidney transplant recipients. At the end of the study, a total of 155 kidney transplant recipients were recruited from October 2015 to October 2017, and classified into acute rejection group (*n* = 34) and stable renal function group (*n* = 121) according to their clinical course. We demonstrate that the serum BAFF levels when acute rejection occurred was significantly higher than that in the stable renal function group (2426.19 ± 892.19 vs. 988.17 ± 485.63 pg/mL, *P* < 0.05). BAFF expression was significantly enhanced in the membrane and cytoplasm of renal tubule epithelial cells in the transplant kidney tissue with acute rejection, and a positive correlation between BAFF and C4d expression was also observed (*r* = 0.880, *P* = 0.001). ROC analyses highlight the superiority of serum BAFF level before transplant over those on other post-transplant days in prediction of acute rejection episodes. The sensitivity, specificity and AUC (area under curve) were 83.3, 89.5, and 0.886%, respectively. Kaplan-Meier survival analysis showed that recipients with higher pretransplant BAFF levels had higher acute rejection incidence (*P* = 0.003). In conclusion, we have identified that BAFF levels are associated with the acute rejection and could be a promising biomarker to predict kidney transplant rejection risks.

## Introduction

The role of B-cells in kidney transplantation has been highlighted in recent studies (1), especially in transplantation tolerance and antibody-mediated rejection (AMR) (2, 3). Besides the well-known effects on humoral immunity, B cells can also uptake, process and present antigens, acting as antigen-presenting cells (APC) to activate and recruit T cells and mediate cellular rejection (4). B cell activating factor (BAFF) belonging to TNF family is a cytokine that enhances B-cell survival and proliferation (3). It participates in many processes involved in B cell regulation, such as activation, proliferation, antibody production and immunoglobulin class switching (5). BAFF is a key factor for the survival of B cells and homeostasis (6). BAFF excess might contribute to the abnormal rescue of self-reactive and might be a key role in B-cell dysregulation (7).

Moreover, BAFF also has a co-stimulatory effect on T cells. Previous study have shown that BAFF-stimulated B cells significantly promoted the proliferation and activity of co-cultured T cells, and increased the percentages of CD4^+^CD28^+^ and CD4^+^CD154^+^ T cells (8). In recent years, the roles of BAFF in transplantation immunity and the induction of transplantation immune tolerance have attracted attentions. However, the relationship between BAFF and allograft rejection is controversial. Several studies demonstrated that BAFF are associated with an increased risk of subsequent antibody-mediated acute rejection (9–11), while there are objections. Slavcev et al. demonstrated that the pretransplant serum concentrations of BAFF in patients with antibody-mediated acute rejection and kidney recipients without rejection did not significantly differ (12). In addition, studies have focused on the relationship between BAFF and AMR, and the role in cell-mediated rejection (CMR) is still unclear.

To further study the question of BAFF involvement in acute rejection in renal transplantation, we conducted this single center prospective study. The aim of this prospective clinical study was to explore the association between BAFF and the occurrence of AR episodes, and whether BAFF can predict acute kidney transplant rejection.

## Materials and Methods

### Patients

This is a single center prospective study that was approved by the Institutional Ethics Committee of the First Affiliated Hospital of Xi'an Jiaotong University. The informed consent was obtained from all study participants. A total of 155 KT recipients were recruited from October 2015 to October 2017, and all were first-time transplant recipients. All kidney grafts were from either living donors or deceased donors after cardiac death. All recipients were confirmed by HLA antibody test and lymphocyte poisoning cross-matching test (CDC) to perform kidney transplantation without HLA antibody to donors. Exclusion criteria: patients were excluded when suffered from infection or delayed graft function during the 6-month follow-up after transplantation. Delayed graft function (DGF) was defined as the requirement of dialysis within 7 days of kidney transplantation.

All patients received induction therapy with anti-human T lymphocyte rabbit immunoglobulin (ATG) and steroids. Subsequent immunosuppressive maintenance regimens were standard triple therapy, which consisted of tacrolimus (FK506) or cyclosporine A (CsA), combined with mycophenolate (MMF) and prednisone.

Patients with stable renal function were defined as those without acute rejections and without an increase >10% in serum creatinine in the last 6 months. The day that serum creatinine levels increased by 25% or more with decreasing urine output, was considered as the day of acute rejection occurred, which was confirmed by the following kidney allograft biopsy. Rejection episodes were treated with methylprednisolone at a dose of 500 mg by intravenous injection once daily for 3 consecutive days. ATG was used in the patients with refractory acute rejection episodes.

### Sample Collection

Non-anticoagulant peripheral venous blood sample and EDTA anticoagulated whole peripheral blood samples were collected from all recipients before transplantation, and at days 7, 14, 21, 28, and at months 2, 3, 4, 5, and 6 after transplantation. Additional blood samples were obtained from AR patients on the day AR occurred.

For the recipients with acute rejection, we carefully read the indications and contraindications for kidney transplant biopsy, made full preoperative preparations, and conducted percutaneous renal biopsy on these patients with the help of B-ultrasound. Two pieces of transplanted kidney tissues were taken. One of them was immediately fixed in 4% formaldehyde for 24 h, and then made into paraffin blocks after dehydration, transparentizing, waxing, and embedding. The other piece was immediately embedded in OCT tissue preservation solution and stored in liquid nitrogen for future use.

### ELISAs

The BAFF levels in patient serum samples were measured by commercially available enzyme-linked immunosorbent assay (ELISA) following the manufacturer's recommended procedures (R&D Systems, Minneapolis, MN). Each sample was tested in duplicate, and the average value is reported as picograms per milliliter (pg/mL).

### RT-PCR

Total RNA was extracted from the peripheral blood mononuclear cells with TRIzol (Invitrogen Life Technologies, Carlsbad, CA), according to the manufacturer's protocol. First-strand complementary DNA (cDNA) was obtained using a reverse transcription system kit (Takara PrimeScript™ RT Master Mix, Takara Biotechnology, Japan). Primers for BAFF and glyceraldehyde-3-phosphate dehydrogenase (GAPDH) (Table 1) was purchased from Takara Biotechnology company (Japan). The mRNA levels of BAFF and GAPDH were measured using the SYBR® *Premix Ex* Taq^TM^ kit (Takara Biotechnology, Japan).

**Table 1:** Primer sequences.

**Gene**	**Primer sequences**
BAFF	F:5′-GAAGCGATAAGTGGAGTCAGTTTCA-3′
	R:5′-CCACATTAGCAGCAACACCAGA-3′
GAPDH	F:5′-GCACCGTCAAGGCTGAGAAC-3′
	R:5′-TGGTGAAGACGCCAGTGGA-3′

*Each sample has three replicates, and their averaged Ct was calculated. The experiment was repeated three times*.

### Histological Analyses

The specimens obtained from biopsy were subjected to pathological diagnosis after embedding, sectioning, and staining. The diagnosis criteria are referred to Banff 2015 criteria (9). The streptavidin-perosidase (SP) linkage method was used to examine the expression levels of BAFF, C4d, and CD20 in transplanted kidney tissues. All the antibodies were purchased from R&D company (R&D Systems, Minneapolis, MN, USA). According to the pathological classification program for transplanted kidney in Banff 2015 (13), the pathological changes of renal tissues were determined by two senior transplant pathologists under microscope in a double-blinded manner. The morphometric parameters for positive BAFF, C4d, or CD20 were determined using a microscope coupled to a computerized morphometry system (OLYMPUS). The Stream professional multi-functional image analysis software was used to quantitatively analyze the images. Five fields were taken from each section: up, down, left, right, and center. The score in each field was calculated, and the section score was represented by the average score from all five fields. The scoring method is as follows: first, according to the proportion of positive cells, assign 0–4 points to 0, 1–25, 26–50, 51–75, and 76–100%, respectively; then, according to the staining intensity of cytoplasm and membrane, assign 0 to 3 points to no staining (–), weak staining (+), medium staining (++), and strong staining (+ + +), respectively. Finally, the two parts were added together to get the total score.

### Statistical Analysis

All data were recorded as the mean± standard deviation. Continuous variables were compared using Student's *t*-test. Categorical variables were compared using the chi-square or the Fisher's exact test. Correlations between the BAFF and other variables were determined using Pearson's correlation coefficients, with correlation expressed as r values. Receiver operator characteristics (ROC) analysis was performed to assess the potential of BAFF for distinguishing patients with and without rejection. All statistical calculations and tests were performed using SPSS 17.0 software (SPSS, Inc., Chicago, IL, USA), GraphPad Prism software (GraphPad Prism Software Inc., San Diego, CA, USA), and MedCalc (MedCalc Software, Mariakerke, Belgium). A *P* < 0.05 was considered statistically significant.

## Results

### Baseline Clinical Characteristics of Study Subjects

A total of 155 kidney transplant recipients who met the inclusion criteria and had complete follow-up data were recruited in the study. There were 108 males and 47 females with an average age of 36.58 ± 13.42 years. At the endpoint of this follow-up, 34 patients experienced AR episodes and 121 patients were categorized into stable renal function group. The occurrence time of acute rejection ranged from 3 to 89 days after surgery, with an average of 32 days after surgery. The general information of each kidney transplant recipients is shown in Table 2. There was no significant difference between the two groups in age, primary disease, pre-operative dialysis, pre-transplant PRA, baseline serum creatinine, type of donor, cold ischemia time, or immunosuppressive regimen (*P* > 0.05). However, statistical difference existed in gender and HLA mismatch (*P* < 0.05).

**Table 2:** The general information of kidney transplant recipients.

**Parameter**	**Stable renal function (*n* = 121)**	**Acute rejection (*n* = 34)**	***P*-value**
Average age (year)	38.72 ± 7.20	33.47 ± 4.30	0.49
Gender (male: female)	89 : 32	19 : 15	0.048
Primary disease			0.27
Glomerulonephritis	109	19	
Diabetic nephropathy	3	1	
Polycystic kidney	4	1	
Anaphylactic purpura nephritis	3	2	
Hypertensive nephropathy	0	1	
Other	2	0	
Pre-operative dialysis			0.11
Hematodialysis	116	30	
Peritoneal dialysis	5	4	
No dialysis	0	0	
Baseline serum creatinine	811.99 ± 264.26	780.67 ± 144.60	0.14
Type of donor			0.31
Living donors	36	8	
Deceased donors after cardiac death	85	26	
Pre-operative PRA			0.12
PRA <10%	120	32	
10% <PRA <30%	1	2	
HLA mismatch			0.01
< = 2 antigenes	69	13	
3 antigens	43	21	
4 antigens	9	0	
Cold ischemia time (h)	3.16 ± 1.85	2.76 ± 0.32	0.06
Immunosuppressive regimen			0.44
CsA+MMF+Pred	29	6	
Fk506+MMF+Pred	92	28	

*PRA, panel reactive antibody; HLA, human leucocyte antigen; CsA, cyclosporin; FK506, tacrolimus; MMF, Mycophenolate Mofetil; Pred, prednisone*.

### Association Between Serum BAFF Levels and Acute Rejection

**Figure 1:**
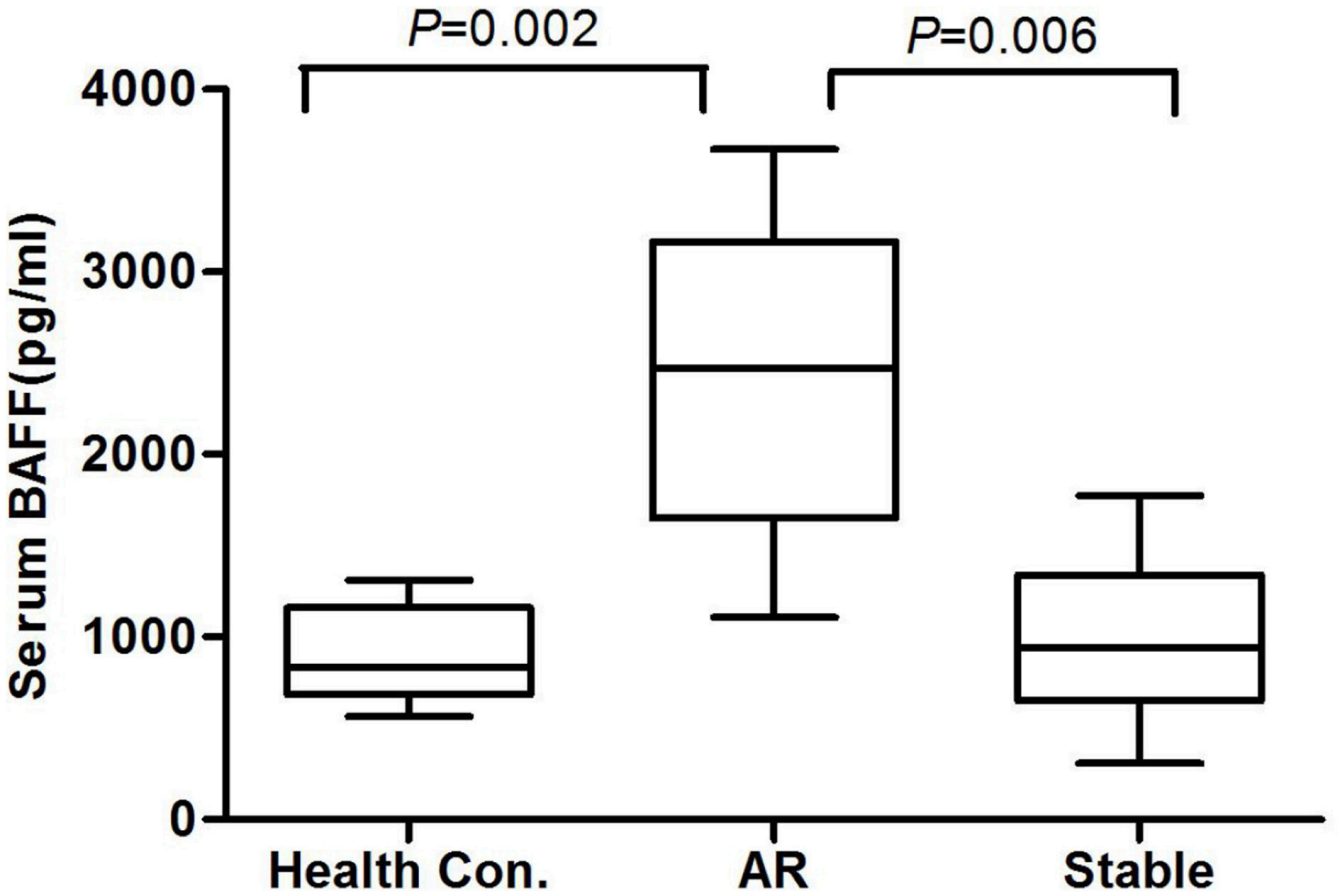
The serum BAFF levels at the time of acute rejection in different groups. The serum BAFF level was 2426.19 ± 892.19 pg/mL (95% confidence interval: 1489.89~3362.49 pg/ml) at the time of acute rejection, significantly higher than that in the stable renal function group (988.17 ± 485.63 pg/mL, *P* < 0.05) and healthy controls (898.10 ± 269.74 pg/mL, *P* < 0.05).

At the end of the 6 months post-transplantation follow-up, 34 patients experienced biopsy-proven AR, among whom, 5 patients suffered from AMR and 29 patients suffered from CMR. Because serum BAFF levels changed dynamically post-transplantation, we used BAFF values of the stable renal function group measured no more than 3 days before or after AR occurrence as the corresponding controls for correlation analysis between BAFF level and AR. By comparison, we found that the serum BAFF level was 2426.19 ± 892.19 pg/mL (95% confidence interval: 1489.89~3362.49 pg/ml) at the time of acute rejection, significantly higher than that in the stable renal function group (988.17 ± 485.63 pg/mL, *P* < 0.05) and healthy controls (898.10 ± 269.74 pg/mL, *P* < 0.05) (Figure 1). Besides, BAFF is elevated in both AMR and CMR, with no statistical significance (2631.91 ± 735.73 vs. 2714.25 ± 875.09, *P* = 0.43).

### BAFF mRNA Expression of Peripheral Blood Lymphocytes

**Figure 2:**
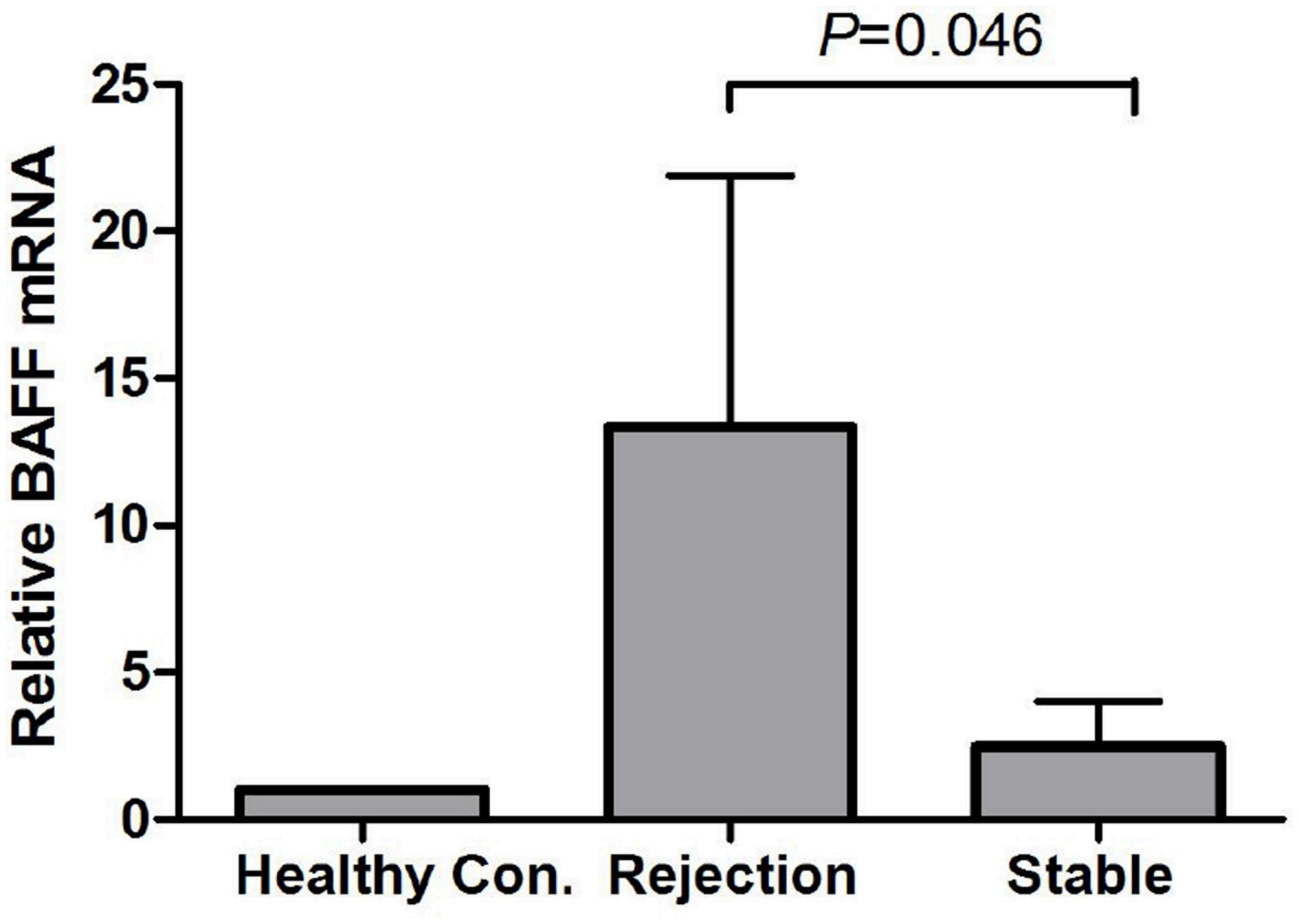
BAFF mRNA levels of peripheral blood lymphocytes during acute rejection. BAFF mRNA level was significantly increased during acute rejection, which was 5.4 times higher than that of stable renal function group.

By using GAPDH gene as the internal reference, we calculated the relative expression of BAFF mRNA with 2-Δt method. Compared with the stable renal function group, BAFF mRNA level was significantly increased during acute rejection, which was 5.4 times higher than that of stable renal function group (Figure 2).

### BAFF Expression in Transplant Kidneys

**Figure 3:**
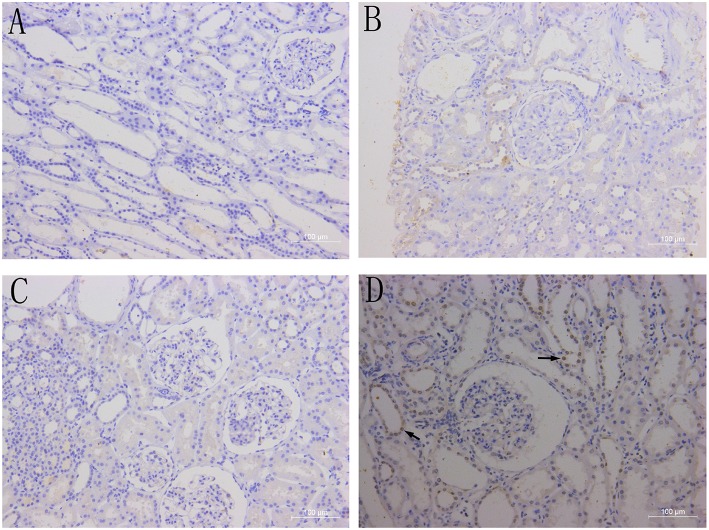
BAFF expression in kidney tissues. Representative images of BAFF staining from healthy controls **(A)** and patients with drug-induced renal damages **(B)**, stable renal function **(C)** or acute cellular rejection **(D)** arrows indicates BAFF-positive staining within the membrane and cytoplasm of renal tubular epithelial cells.

There was no BAFF staining in the kidney tissues of healthy control (Figure 3A), and no or weak expression in the transplanted kidney tissues with drug-induced renal damages (Figure 3B) and stable renal function underwent protocol biopsy (Figure 3C). However, in the transplant kidney with either AMR or CMR (Figure 3D), BAFF expression was significantly enhanced in the membrane and cytoplasm of renal tubule epithelial cells, as well as the inflammatory cells infiltrated between glomeruli and renal tubules.

### Correlations Between BAFF, CD20, and C4d Expression in Transplant Kidneys

**Figure 4:**
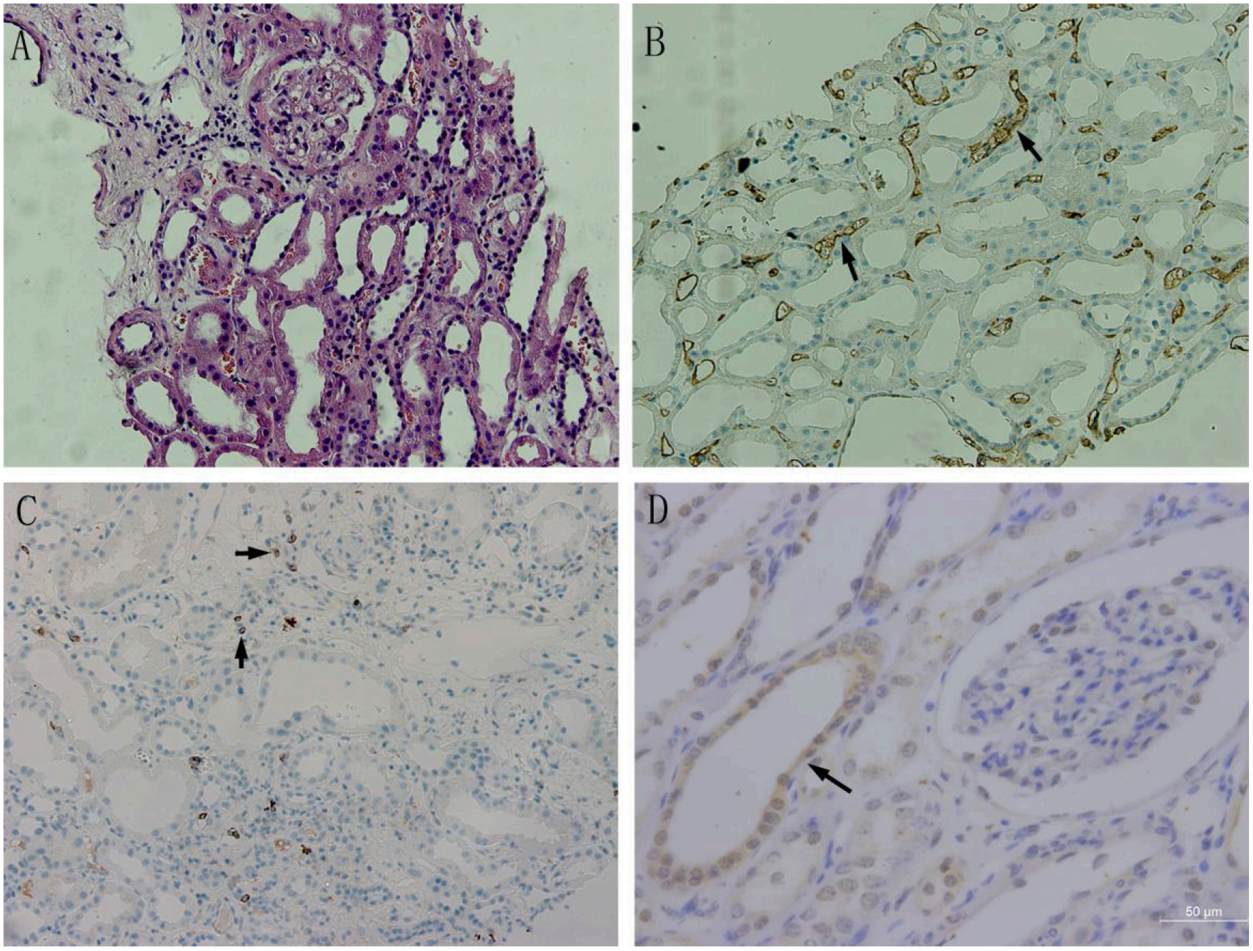
The BAFF expression in kidney tissues with acute rejection. **(A)** Acute humoral rejection (HE, ×200): swollen glomerular endothelial cells, degenerated tubular epithelial cells,shed brush borders, and mononuclear lymphocytes and erythrocyte deposits in the perivascular capillaries. **(B)** C4d expression in transplant kidney tissue (×200): C4d was strongly and linearly deposited at perivascular capillary basal membranes, and its deposition was also observed in the glomerular capillary lumen. **(C)** CD20 expression in transplant kidney tissue (×200):CD20-positive B lymphocytes were mainly accumulated at renal interstitium, exhibiting dispersive infiltration. **(D)** BAFF expression in transplant kidney tissue(×400). BAFF was mainly expressed on the membrane and cytoplasm of renal tubular epithelial cells.Positive staining was observed as a brown color.

BAFF was mainly expressed on the membrane and cytoplasm of renal tubular epithelial cells. In addition, BAFF expression was also seen on the membrane and cytoplasm of inflammatory cells that infiltrated between glomeruli and renal tubules. CD20-positive B lymphocytes were mainly accumulated at renal interstitium, exhibiting dispersive infiltration. C4d was strongly and linearly deposited at perivascular capillary basal membranes, and its deposition was also observed in the glomerular capillary lumen (Figure 4).

According to the evaluation and scoring criteria for immunohistochemistry results, as described above, we calculated the immunohistochemistry score of each renal tissue section from acute rejection group, and semi-quantitatively analyzed the results. Pearson correlation was used to analyze the relationships between the expression levels of BAFF, CD20, and C4d in transplanted kidney tissues. The results showed that there was a positive correlation between BAFF and C4d expression in transplanted kidney tissues (*r* = 0.880, *P* = 0.001), but no correlation was found between CD20 and BAFF expression (*r* = 0.609, *P* = 0.062), or between C4d and CD20 expression (*r* = 0.356, *P* = 0.313) (Table 3).

**Table 3:** The correlation between BAFF, CD20, and C4d expression levels in transplanted kidneys.

**Gene**	**BAFF**	**C4d**
BAFF		*r* = 0.880(*P* = 0.001)
CD20	*r* = 0.609(*P* = 0.062)	*r* = 0.356(*P* = 0.313)

### Serum BAFF Level Predicting Acute Rejection in Kidney Transplant Recipients

**Figure 5:**
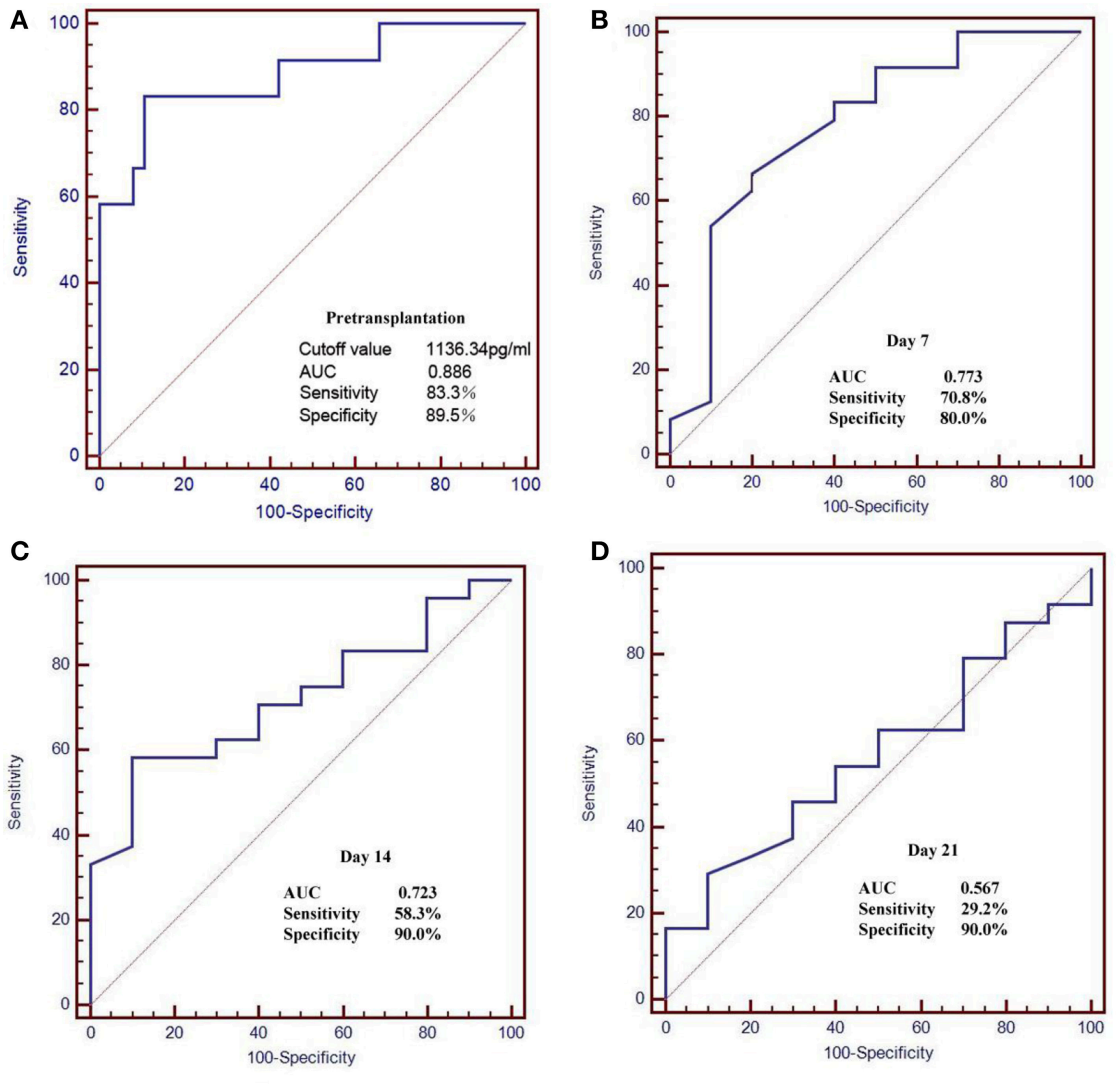
Receiver operating characteristic (ROC) curves for serum BAFF level in the prediction of AR. **(A)** The pretransplant serum BAFF levels presented the best predictive value with the biggest areas under the curve (AUCs) (0.886), and showed a good sensitivity (83.3%) and specificity (89.5%). **(B–D)** The serum BAFF on day 7, 14, 21 post transplant presented lower sensitivity and specificity than the pretransplant serum BAFF.

The pre-transplant serum BAFF level was higher in acute rejection group compared to the stable renal function group (1517 ± 441.24 vs. 904.82 ± 236.87 pg/mL, *P*<0.05). We used receiver operating characteristic (ROC) curves to analyze the sensitivity and specificity of pretransplant serum BAFF level and the value at days 7, 14, 21 after transplantation in predicting acute rejection. The pretransplant serum BAFF levels presented the best predictive value with the biggest areas under the curve (AUCs) (0.886), and showed a good sensitivity (83.3%) and specificity (89.5%) (Figure 5).

**Figure 6:**
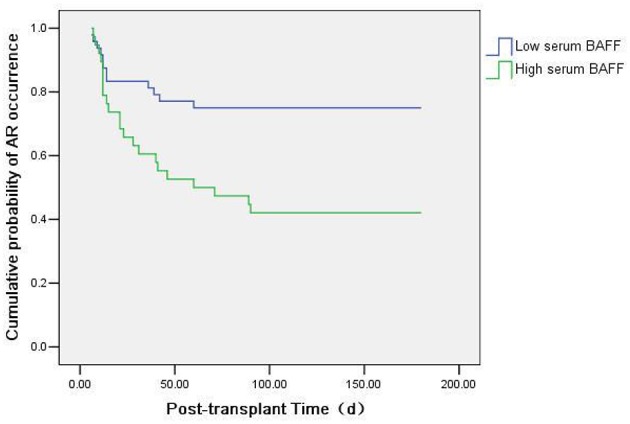
Kaplan-Meier survival analysis of the relationship between preoperative serum BAFF level and acute rejection. The incidence of acute rejection was higher in the high serum BAFF group than in the low serum BAFF group (*P* = 0.003).

We also used Kaplan-Meier survival analysis to compare the acute rejection occurrence in kidney transplant recipients with high serum BAFF level (>1136.34 pg/mL) and low serum BAFF level (BAFF < 1136.34 pg/mL). The result showed that the incidence of acute rejection was increased in the high serum BAFF group than in the low serum BAFF group (*P* = 0.003) (Figure 6).

## Discussion

Recent reports has demonstrated that BAFF is associated with acute antibody-mediated rejection (14) and allograft survival (15, 16), and might be a predictor of antibody mediated rejection in kidney transplantation recipients (11). Although inconsistent results still need to be clarified, Gemma B et al. observed that elevated BAFF concentrations were associated with an increased risk of AMR in recipients with donor-specific antibody (DNA). In contrast, the risk of AMR was not observed in recipients without DSA (9). Slavcev et al. claimed no correlation was found between BAFF and the production of donor-specific antibodies before and after transplantation, and the serum concentrations of BAFF in patients with AMR and kidney recipients without rejection did not significantly differ (12). In addition to these controversies, the clinical significance of BAFF in predicting rejection is not well-understood. In our study, we suggest that the BAFF could be a risk factor for the development of acute rejection in kidney transplant recipients, and be a promising biomarker for non-invasive predicting and diagnosis of acute rejection.

In this study we detected both membrane-bound form and soluble form of BAFF by ELISA and real-time fluorescent quantitative PCR, respectively, and we revealed that both forms were significantly increased when acute rejection occurred. The results demonstrated a relation between the BAFF and acute rejection in kidney transplantation recipients. Previous studies have shown elevated BAFF levels in B lymphocyte clearance, and inflammatory conditions (17–19). Ye Q et al. found that the BAFF-specific receptor BAFF-R was expressed on the surface of CD4^+^ T lymphocytes, and the BAFF knockout mice survived longer than the wild type mice under allogeneic heart transplantation, suggesting that the BAFF/BAFF-R pathway played an important role in the activation and proliferation of T lymphocytes. By blocking the BAFF/BAFF-R pathway, the rejection of allografts can be alleviated (20). In addition, BAFF can promote the secretion of IL-10 and other cytokines by B cells, and negatively regulate T cell function in some aspects (21). The results obtained from this study suggest that the BAFF signaling pathway plays an important role in both AMR and CMR in kidney transplantation immune response, and deserves further study.

There are a few studies had suggested the positive BAFF deposition in kidney tissue in autoimmune disease (22, 23). In this study we detected the BAFF expression within kidney during acute rejection episodes. We found that no BAFF expression was found in kidney tissues of healthy controls, and only weak BAFF expression was found in the kidney tissues with drug-induced renal damage. However, BAFF expression was significantly enhanced in the kidney tissues with acute rejection either AMR or CMR, suggesting that BAFF might participate in the processes of transplantation immune response. Because the well-known important functions of BAFF for the survival and maturation of B lymphocytes, the role of BAFF in AMR has been reported repeatedly, while it is not clear in patients with CMR. Interestingly, we found that BAFF expression was increased in renal tissues with CMR. This finding suggests that BAFF might participate in the processes of CMR development. The specific mechanism may be related to the co-stimulatory effect of BAFF on T cells which has been confirmed in studies of autoimmune diseases. Christine et al. reported BAFF signaling through BR-3 promoted T follicular helper cells accumulation in lupus-prone mice (24). Unfortunately, there are only a 5 cases of AMR in our study. Statistical calculation is limited in comparing the difference of BAFF expression between AMR and CMR due to the small sample size.

In this study, we performed immunohistochemistry to examine the expression of BAFF, C4d, and CD20 in transplant kidney tissues with acute rejection. The results showed a positive correlation between BAFF and C4d expression. Similar to the distribution of C4d, BAFF was also mainly expressed in the cytoplasm and membrane of peripheral tubular epithelial cells. The deposition of C4d in peritubular capillaries has become an important criterion for the diagnosis of humoral rejection (25). Therefore, considering the correlation between BAFF and C4d expression in acute rejection, and the roles of BAFF in B lymphocyte activation, proliferation, and antibody production, we believe that BAFF, to some extent, participates in the antibody-mediated humoral rejection. In addition, no correlation was found between BAFF and CD20 expression in transplanted kidney tissues, suggesting that the role of BAFF in humoral rejection is not dependent on the number of B cells. Consistent to this observation, Bloom et al. used CD52-specific antibody Alemtuzumab to treat humoral rejection after renal transplantation, and found that there was no significant correlation between elevated BAFF levels and the number of B-cell clones induced by BAFF (26). When confronted with literature data, some studies reported that BAFF has a good correlation with DSA (15). We assumed that BAFF might be a biomarker in the diagnosis of AMR together with C4D and DSA. Further large-scale multi-center studies are needed in the future to confirm this hypothesis.

Furthermore, our results showed that, with the increase of pre-transplant serum BAFF levels, the incidence of acute rejection after renal transplantation also increased. The pre-transplant serum BAFF level in acute rejection group was significantly higher than that in the stable renal function group. ROC curve analysis showed that the pre-transplant serum BAFF level could well-predict the risk of acute rejection, with the sensitivity of 83.3% and specificity of 89.5%. Moreover, the incidence of acute rejection in higher serum BAFF group was greater than the serum lower BAFF group. These results indicate that pre-transplant serum BAFF levels can be used as a biomarker to assess the immune status of renal transplant recipients and to predict the risk of postoperative acute rejection. Previously, Xu et al. also found that the BAFF expression from peripheral blood mononuclear cells was significantly elevated in recipients with abnormal renal function compared with the recipients with normal renal function (27). These data indicate that serum BAFF levels can, at least partially, predict the risk of acute rejection after kidney transplantation.

In this study, we confirmed the relationship between BAFF and acute rejection in kidney transplant recipients. We demonstrated that patients with higher pretransplant BAFF levels had an increased acute rejection incidence, and BAFF could be a promising biomarker to predict kidney transplant rejection risks. Furthermore, our results might help stratify the “immunological risk” to better adapt the strategy of prevention in transplanted patients. However, further clinical validation and translational medicine research is necessary to convince the availability from bench to bedside. However, there are still some weaknesses in our study. In order to explore the relationship between serum BAFF level and the donor-specific antibodies (DSA) newly generated in renal transplant recipients, we also dynamically monitored the DSA in all recipients, hoping to study the role of BAFF in antibody-mediated humoral rejection from another perspective. However, due to the small sample size, the statistics did not meet the requirements. In the future, we will further increase the sample size, and perform deeper investigations on the role of BAFF in acute rejection.

## Ethics Statement

This study was carried out in accordance with the recommendations of Institutional Ethics Committee of the First Affiliated Hospital of Xi'an Jiaotong University with written informed consent from all subjects. All subjects gave written informed consent in accordance with the Declaration of Helsinki. The protocol was approved by the Institutional Ethics Committee of the First Affiliated Hospital of Xi'an Jiaotong University.

## Author Contributions

X-ZW and ZW performed the experiments. W-JX and JZ designed the experiments. YL interpreted the data. X-ZW and ZW drafted the manuscript. All the authors approved of the final version for publication.

### Conflict of Interest Statement

The authors declare that the research was conducted in the absence of any commercial or financial relationships that could be construed as a potential conflict of interest.
